# Chemogenetic activation of cholinergic intrinsic cardiac ganglia improves border zone oxygenation and reduces arrhythmias during acute local ischemia

**DOI:** 10.3389/fcvm.2026.1814067

**Published:** 2026-06-09

**Authors:** Bridget R. Alber, Aman Gill, Jhansi Dyavanpalli, David Mendelowitz, Matthew W. Kay

**Affiliations:** 1 Department of Biomedical Engineering, The George Washington University, Washington, DC, United States; 2 Department of Pharmacology and Physiology, The George Washington University, Washington, DC, United States

**Keywords:** acute myocardial infarction, chemogenetics, intrinsic cardiac ganglia, myocardial ischemia, oxygenation, parasympathetic nervous system

## Abstract

**Background:**

Current therapeutic strategies for acute myocardial infarction rely on reperfusion and pharmacological management, which are typically administered hours after an event. Activation of the cardiac cholinergic efferents superior to the heart soon after a coronary occlusion has shown promise as a potential therapy to reduce arrhythmias and improve ventricular function. We tested whether selective activation of cholinergic neurons within the intrinsic cardiac ganglia (ICG) would also reduce arrhythmias and improve oxygenation of ischemic border zone tissue after an acute coronary occlusion.

**Methods:**

Designer Receptors Exclusively Activated by Designer Drugs (DREADDs) were selectively expressed in cholinergic neurons of the ICG via pericardial sac injections of an HM3Dq DREADDs virus in transgenic rats that expressed Cre recombinase in cholinergic neurons. Cholinergic ICG neurons were activated using the synthetic DREADDs ligand clozapine-N-oxide (CNO). Heart rate reductions after intraperitoneal injection of CNO confirmed downstream effect of DREADDs-mediated cholinergic ICG activation in ECG telemetry studies. The effect of cholinergic ICG activation on PR interval, arrhythmia burden, ischemic border zone tissue oxygenation and epicardial NADH fluorescence 20 min after ligation of the left anterior descending coronary artery (LAD) was then studied in excised perfused hearts of DREADDS-expressing rats and rats that did not receive the HM3Dq DREADDs virus.

**Results:**

LAD ligation resulted in a well defined ischemic zone that encompassed a large portion of the left ventricle, where pO2 in the center of the ischemic zone typically dropped to 0 mmHg within 10 s. Subsequent DREADDs-mediated cholinergic ICG activation prolonged the PR interval from 39.13 ± 6.17 ms to 42.46 ± 6.87 ms and lowered the incidence of arrhythmia from 0.9398 ± 0.5063 $\min^{-1}$ to .5727 ± 0.3103 $\min^{-1}$. DREADDs-mediated cholinergic ICG activation also increased ischemic border zone pO2 from 42.13 ± 49.82 mmHg to 82.25 ± 66.87 mmHg and NADH fluorescence trended lower in the ischemic zone, indicating increased mitochondrial oxidation. These effects were blocked when the muscarinic antagonist atropine was administered before CNO.

**Conclusion:**

Results indicate that selective stimulation of cholinergic ICG neurons could improve local delivery of oxygen to the ischemic border zone soon after a coronary occlusion and reduce arrhythmia burden through a muscarinic-dependent mechanism, supporting further studies of the intrinsic cardiac cholinergic network as a therapeutic target for early intervention before reperfusion therapy to activate cardioprotective pathways.

## 1 Introduction

Cardiovascular disease (CVD), including myocardial infarction (MI), remains a major health concern worldwide [1]. Coronary occlusion triggers a series of pathological changes that include cardiomyocyte death, inflammation, and remodeling that contribute to ventricular dysfunction and arrhythmias [2, 3]. The first hour after an acute MI is a critical period with high incidence of life-threatening arrhythmias that significantly contribute to early mortality [4]. Limited perfusion to the infarct core restricts therapeutic access; however, collateral circulation could improve myocardial viability in the infarct border zone [5, 6]. This border zone region represents a therapeutically accessible target for interventions aimed at reducing acute arrhythmias and improving outcomes.

The autonomic nervous system regulates cardiac function and modulates physiological responses to myocardial injury [7]. Many disease states including hypertension, heart failure, and arrhythmias exhibit sympathetic hyperactivity and diminished parasympathetic tone [8]. While beta-blockers effectively reduce sympathetic overdrive after MI, directly enhancing cardiac parasympathetic activity may provide additional complementary cardioprotection [9]. Vagal stimulation during or after an MI has been shown to reduce arrhythmia incidence and provide cardioprotective effects [10], in part by improving autonomic balance [11] and decreasing ventricular excitability in infarct border zone [12]. In a clinical study, patients with ST-elevation MI treated with low-level tragus stimulation showed reduced ventricular arrhythmias during the first 24 h and improved cardiac function [13]. A major limitation of current approaches to activate cardiac parasympathetic neurons is a lack of cardiac specificity, resulting in systemic effects that may limit clinical efficacy [14].

Cell-specific activation of the cardiac parasympathetic neurons is a promising new approach to treat cardiac disease. The challenge of targeting the cardiac neurons is the cellular heterogeneity of the intrinsic cardiac nervous system. This heterogeneity involves parasympathetic neurons, sympathetic postsynaptic neurons, interneurons, sensory neurons, and various non-cholinergic nerve inputs [15, 16]. Consequently, electrical stimulation of the intrinsic cardiac ganglia (ICG) would non-selectively activate both parasympathetic and other neuronal populations [17]. Addressing this limitation, cardiac-specific optogenetic vagal stimulation in a mouse model showed improved cardiac function after MI [18], demonstrating the therapeutic potential of selectively targeting parasympathetic neurons. Previous work has also shown that daily chemogenetic activation of a specific population of neurons in the paraventricular nucleus (PVN) of the hypothalamus that co-release oxytocin and glutamate to excite the cardiac vagal cholinergic neurons improves cardiac outcomes and reduces arrhythmias 7 days after MI [19]. Within the heart, chronic cholinergic ICG stimulation also improved cardiac outcomes in an 8-week model of pressure overload heart failure [20]. Similar to that prior study, we used the same Cre-lox approach to selectively express Designer Receptors Exclusively Activated by Designer Drugs (DREADDs) within cholinergic ICG neurons in transgenic rats having a choline acetyltransferase (ChAT) promoter of Cre expression.

Although daily cholinergic neuron stimulation provides significant long-term benefits during heart failure and after MI, delivering timely interventions soon after myocardial injury also has the potential to reduce long-term damage. The period soon after MI presents a distinct and critical therapeutic window where targeted activation of cholinergic neurons could activate beneficial muscarinic pathways that may promote vasodilation to increase collateral flow within border zone tissue and decrease arrhythmia incidence by suppressing adrenergic pathways and Purkinje fiber automaticity. We therefore tested the hypothesis that selective activation of cholinergic ICG neurons would improve tissue oxygenation in the ischemic border zone and reduce arrhythmias after acute left anterior descending artery (LAD) ligation.

## 2 Methods

All animal procedures were completed in agreement with The George Washington University institutional guidelines and in compliance with the panel of Euthanasia of the American Veterinary Medical Association and the National Institutes (NIH) Guide for the Care and Use of Laboratory Animals.

### 2.1 ChAT-cre rats and selective expression of DREADDs

Adult male and female Long Evans (LE) transgenic ChAT-Cre rats (8–10 weeks old) were used for all experiments. Hemizygous LE-Tg(ChAT-Cre)5.1Deis rats, obtained from Rat Resource & Research Center (Columbia, MO), were bred to produce offspring that expressed Cre recombinase in cholinergic neurons. Four day old pups received pericardial sac injections of HM3Dq DREADDs virus (AAV2-hSyn-DIOhM3D(Gq)-mcherry) to target DREADDs expression to the ICG. Pups were anesthetized by hypothermia for approximately 20 min until the absence of pedal reflex was confirmed. Once anesthetized, the pup was placed supine on a sterile field on top of an ice pack and a small skin incision was made between the 3rd and 4th ribs. Two sterilized 0.5 mm stainless steel forceps were used to blunt dissect the muscle between the ribs, exposing the heart. A pair of fine forceps gently lifted the pericardial sac while a Hamilton syringe (7635-01) with a 34-gauge needle was used to pierce the pericardial sac and inject 2.5 microliters of DREADDs virus. The ribs were sutured together using a single stitch of 6-0 resorbable vicryl suture (Ethicon Vicryl Suture, 6-0, RB-1). The skin incision was closed with 1-3 stitches of 6-0 silk suture (Ethicon Permahand Silk Suture, 6-0, P-1). The pup was then placed on a heating pad, and once warm and responsive, was returned to the mother. Opioid analgesic was administered with a single dose of subcutaneous extended release buprenorphine (Ethiqa XR) at 0.65 mg/kg body weight. Pericardial sac injections for rats in the DREADDs group contained the DREADDs virus while injections for rats in the control no DREADDs group contained only saline. Both groups received the synthetic DREADDs ligand clozapine-N-oxide (CNO) to control for off-target effects including the potential back-conversion of CNO to clozapine.

To further confirm DREADDs expression in DREADDs-expressing rats, the hearts of a subset of rats were excised for confocal imaging of mCherry expression. The ICG of those hearts was dissected and imaged (Leica TCS SP8 MP) with a x40 water immersion lens [20].

### 2.2 Telemetry implant surgery

Six-week old rats were anesthetized with 2% isoflurane in a plexiglass chamber to achieve a surgical plane of anesthesia and were placed on a sterile, warmed surgical field where they were transferred to a nose cone that continued to administer 2% isoflurane, maintaining a surgical plane of anesthesia throughout the procedure. The fur near the incision sites was shaved, and the skin was sterilized with ethanol and chlorhexidine (DYNA-HEX 4). A 2 cm midline incision was made from the lower thorax to the abdomen, followed by a straight incision along the linea alba, with blunt dissection performed laterally. An ETA-F10 transmitter (Data Sciences International) was placed ribs-up in the abdominal cavity with leads facing downward. An 18G needle was used to tunnel the leads through the muscle wall and then they were fed subdermally toward the chest with forceps. The silver lead was sutured to the right pectoral region and the red lead was sutured 1 cm lateral to the xiphoid process at the end of the costal arch. Excess lead length was placed in the abdominal cavity. The abdominal cavity was closed using resorbable 4-0 suture (Oasis, Violet Braided Polyglycolic Acid Absorbable Suture, MV-J315) and the skin was closed with non-resorbable 4-0 silk suture (Oasis, Silk, Non-Absorbable Suture, MV-629-V). Extended release buprenorphine (Ethiqa XR) was administered at 0.65 mg/kg body weight in combination with meloxicam (Covetrus).

### 2.3 Telemetry recordings

Rats were acclimated to the recording environment for one week before data collection. On the day of recording, rats underwent a 30 min acclimation period before transmitters were turned on. ECG signals were recorded using EMKA’s telemetry system, allowing continuous ECG monitoring in freely moving rats. Baseline Heart rate (HR) was recorded for 15 min followed by an intraperitoneal (IP) injection of either CNO (1 mg/kg) or saline. ECG recordings continued for an additional 75 min post-injection. HR was analyzed using LabChart and the baseline HR was averaged over the first 15 min of recording and the HR after CNO was averaged over the last 30 min of recording.

### 2.4 Excised perfused hearts

Rats were anesthetized with 5% isoflurane inhalation in a plexiglass chamber to provide a deep plane of anesthesia until pedal reflex was absent then placed supine on a surgical field with a nose-cone delivering 5% isoflurane to maintain a deep plane of anesthesia. The heart was then excised and immediately placed in cold Krebs-Henseleit (KH) buffer solution supplemented with heparin. The aorta was secured onto a 14G catheter (Terumo Surflo IV Catheter, 14G, SROX1451CA) with size 0 silk suture (LOOK, 0 Silk Suture Spool, SP119) and flushed with cold KH solution containing (in mM) 118 NaCl, 4.7 KCl, 1.25 ${\mathrm{CaCl}}_{2}$, 0.57 ${\mathrm{MgSO}}_{4}$, 1.17 ${\mathrm{KH}}_{2}{\mathrm{PO}}_{4}$, 25 ${\mathrm{NaHCO}}_{3}$, and 6.0 glucose. The heart was then transferred to the perfusion system and retrograde perfused at a constant hydrostatic pressure of 75 mmHg with 37 ${}^{\circ }$C KH oxygenated with 95% $O_{2}$ and 5% ${\mathrm{CO}}_{2}$.

### 2.5 Measurement of tissue oxygenation and NADH imaging

Local tissue oxygenation (${\mathrm{pO}}_{2}$, mmHg) and temperature were measured at a site within the lateral left ventricle (LV) halfway between the apex and the base using a small diameter (450 μm) optical probe (Oxford Optronix OX-NX-BF/O/E). The oxygen probe was inserted into a track created within the ischemic border zone using a 25G needle. Epicardial NADH fluorescence (fNADH), a rapid label-free readout of mitochondrial redox state [21], was imaged to monitor the size and location of the ischemic zone after occluding the LAD [22]. This enabled the oxygen probe to be positioned at the border between ischemic and non-ischemic tissue while also providing a detailed assessment of any changes in the distribution of fNADH within the ischemic zone during the experimental protocol. Epicardial NADH was excited using two 365 nm LED lights, and the emitted autofluorescence was bandpass filtered at 475 nm (Chroma HQ475/50) and imaged using a CCD camera (Andor iXON Life 897).

### 2.6 Experimental protocol

**Figure 1:**
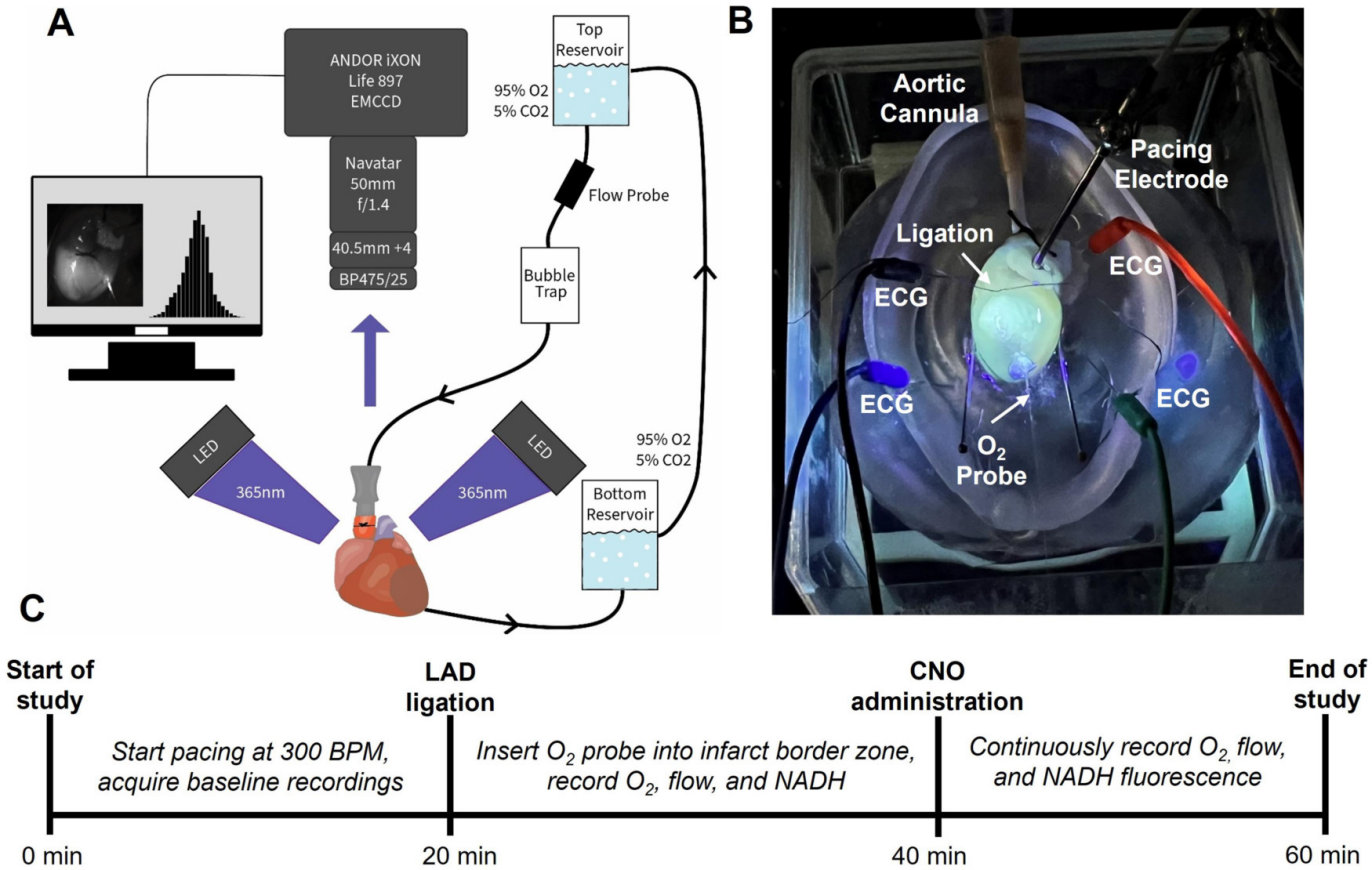
Constant pressure perfusion system and experimental timeline. **(A)** Schematic illustration of the Langendorff heart perfusion system and the placement of UV LEDs (365 nm) and the CCD camera to image epicardial NADH fluorescence. **(B)** Photograph of a perfused heart in the warm bath of perfusate surrounded by four ECG electrodes and paced with a concentric pacing electrode. An Oxylite $O_{2}$ probe is positioned within the border of the ischemic zone to measure ${\mathrm{pO}}_{2}$ simultaneously with NADH fluorescence. **(C)** Timeline of the experimental protocol.

There were a total of 25 animals used for the experiments, 8 No-DREADDs (5M, 3F), 9 DREADDs (5M, 4F), and 8 DREADDs+Atropine (4M, 4F). There was no statistically significant difference between males and females in any of the parameters measured. Hearts were equilibrated for 5–10 min after initiating perfusion. To control for the effect of HR on measured outcomes, hearts were paced at 5 Hz (300 BPM, 3.5 mA, 5msec pulse duration) using an electrode placed on the surface of the left atrium. Pacing was interrupted for 1 min every 5 min to assess arrhythmia burden. The LAD was then permanently ligated with 6-0 silk suture (Ethicon Permahand Silk Suture, 6-0, P-1). 20 min after LAD ligation, 10 μM circulating CNO was administered as a bolus dose into the aortic inflow line directly above the aorta. The ECG, coronary flow, and ${\mathrm{pO}}_{2}$ were simultaneously acquired (1,000 samples/sec) using a PowerLab unit and LabChart software (ADinstruments), concurrently with epicardial fNADH for 20 min after CNO administration (Figure 1C). In a subset of studies, the muscarinic antagonist atropine (50 nM circulating concentration) was administered to the top reservoir of the perfusion system before administering CNO to confirm that blocking muscarinic receptors prevents the effects of DREADDs activation of cholinergic ICG neurons.

### 2.7 Myocardial area-at-risk

The size of the ischemic zone was assessed at the end of each study by retrograde perfusing hearts with 10 mL of 1% Evans Blue dye to delineate perfused and non-perfused regions. Hearts were then transversely sectioned (2 mm thick) at the base, midwall, and apex to assess the transmural extent of the ischemic zone. Sections were incubated in 42 mM 2,3,5 -Triphenyltetrazolium chloride (TTC) solution at 37 ${}^{\circ }$C for 10 min with gentle agitation. TTC stains metabolically active tissue red. Co-staining with Evans Blue and TTC identified perfused healthy tissue as purple. Unperfused non-metabolically active tissue did not change color. Sections were then photographed to measure the myocardial area-at-risk as the size of the unperfused non-metabolically activate tissue using a custom Matlab script.

### 2.8 Data analysis

The ECG, coronary flow, and ${\mathrm{pO}}_{2}$ signals were viewed and analyzed using LabChart software. Coronary flow was averaged over 5 min before and 5 min after LAD ligation. Baseline ${\mathrm{pO}}_{2}$ was averaged from the time the probe stabilized until CNO administration. Post-CNO ${\mathrm{pO}}_{2}$ was averaged over the 20 min following CNO administration. PR-interval was calculated in LabChart immediately before and 10 min after CNO administration. Arrhythmia incidence was assessed by manual review of ECG signals of each heart during four one-minute intervals of no pacing. Arrhythmia counts were averaged per minute. The ECG reviewer was blinded to the animal ID and the experimental group. A premature ventricular contraction (PVC) was defined as a premature QRS complex of unique morphology that lacked a preceding p-wave. Non-sustained ventricular tachycardia (NSVT) was defined as a sequence of more than three rapid beats of consistent unique morphology that terminated within 10 s. Ventricular fibrillation (VF) was identified as a sustained rapid irregular rhythm having indiscernible P-QRS-T waves. fNADH images were analyzed using custom Matlab scripts. Pixel-by-pixel fNADH differences were calculated between the average of the first 100 frames and the average of the final 100 frames over a 10 min period. Pixel-wise changes were binned into histograms for further analysis, and the Gaussian means were compared.

### 2.9 Statistical analyses

**Figure 2:**
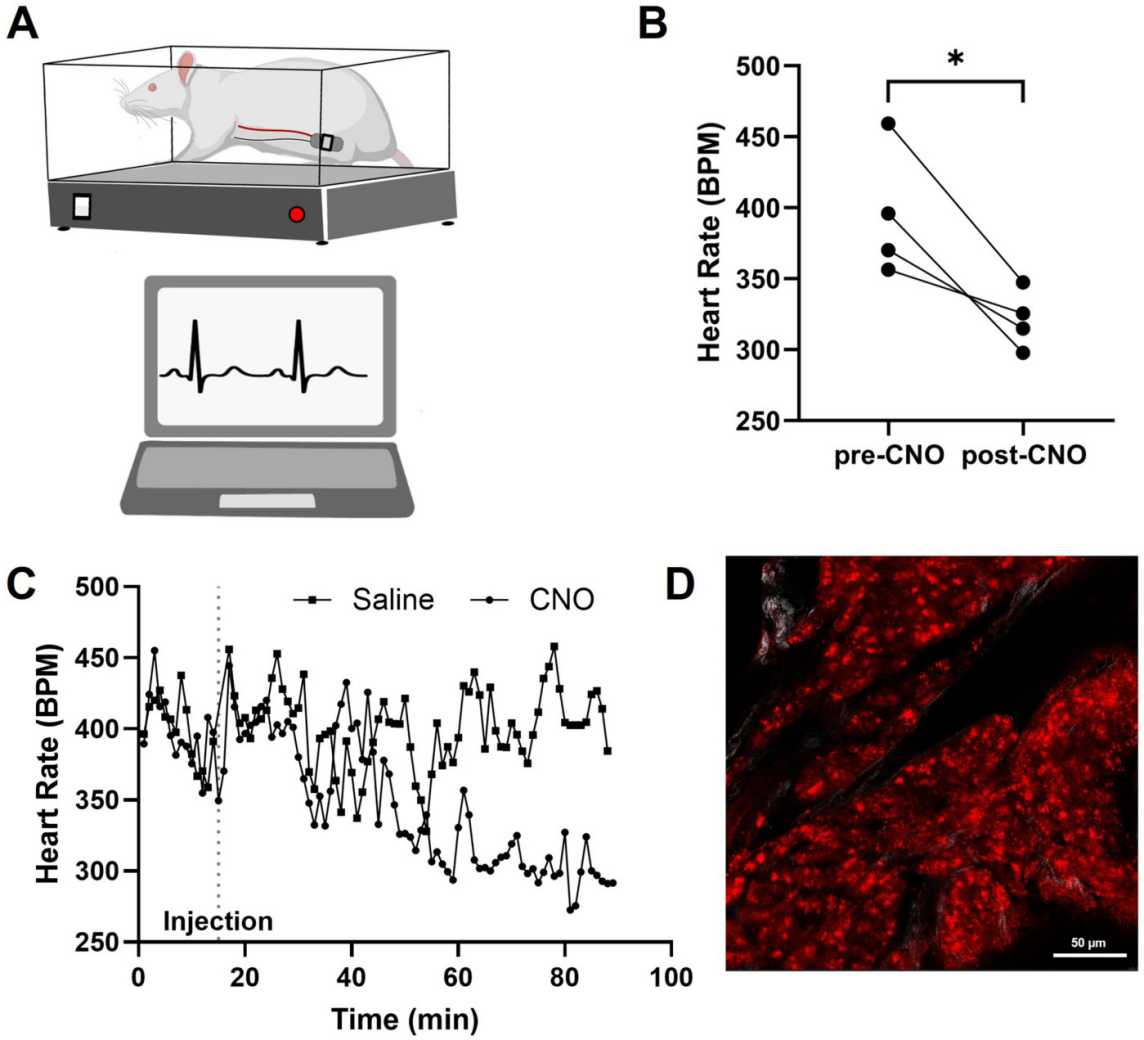
*In vivo* DREADDs activation of cholinergic ICG neurons reduces heart rate. **(A)** Schematic representation of the experimental setup for HR monitoring in unrestrained Chat-Cre Long Evans rats with an implanted DSI ETA F-10 transmitter. **(B)** HR before and after CNO administration in DREADDs-expressing rats. A significant reduction in HR is observed after CNO treatment (two-tailed paired *t*-test, *p* = 0.029, *n* = 4). **(C)** Representative time course of HR following administration of saline (top) or CNO (bottom) in the same DREADDs-expressing rat during two separate recordings. HR dropped after CNO administration compared to saline administration. **(D)** Confocal image showing expression of DREADDs in the ICG. Red indicates DREADDs-linked-mCherry fluorescence.

Data were processed, analyzed, and graphed using GraphPad Prism 10. Data were reported as mean ± SD and graphed as mean ± SEM in the figures. After confirming normality and equal variance, measurements between groups were compared using unpaired or paired two-tailed *t*-tests, while multiple-group comparisons were analyzed using one-way ANOVA with Tukey’s post hoc comparison. The nonparameteric Kolmogorov-Smirnov test was used to compare datasets that were not normal, as indicated in the figure captions. A *p*-value <0.05 was considered statistically significant. The experimental complexity of the *in vivo* and *ex vivo* measurements resulted in an unequal number of assessments for each group of animals. The primary causes of differences in sample sizes were the limited availability of transmitters for the *in vivo* studies (Figure 2), ECG signals with low signal to noise ratios that prohibited accurate detection of PR intervals (Figure 4), and optical glare during epicardial imaging of fNADH (Figure 6).

## 3 Results

Rats with Cre-dependent excitatory HM3Dq receptor selectively expressed within the ICG [20] were studied to test the hypothesis that activation of the intrinsic cholinergic neurons would improve tissue oxygenation within the ischemic border zone and reduce arrhythmia incidence after an acute MI. *In vivo* telemetric ECG recordings of those rats confirmed cholinergic reductions in HR after HM3Dq receptor activation with CNO. The LAD of excised perfused hearts from those rats was ligated to mimic an MI, resulting in an overall drop in coronary flow and tissue oxygenation, with ST-segment elevation and tissue staining to confirm a large transmural ischemic zone. Results indicated that acute activation of HM3Dq receptors with CNO after LAD ligation improved oxygenation of the ischemic border zone and reduced the incidence of arrhythmias.

### 3.1 Cholinergic ICG activation slows HR *in vivo*

HR dropped over one hour in DREADDs-expressing rats after a CNO injection (Figure 2), confirming functional DREADDs expression and downstream cholinergic slowing of HR. HR dropped from approximately 400 BPM to 300 BPM after CNO administration in one DREADDs-expressing animal with no apparent change following saline injection (Figure 2C). On average, HR significantly dropped from baseline (395.4 ± 45.60 BPM) to post-CNO administration (321.4 ± 20.77 BPM; *p* = 0.029, *n* = 4) (Figure 2B). Confocal imaging of HM3Dq-mCherry fluorescence further confirmed DREADDs expression in the ICG (Figure 2D).

### 3.2 Local ischemia

**Figure 3:**
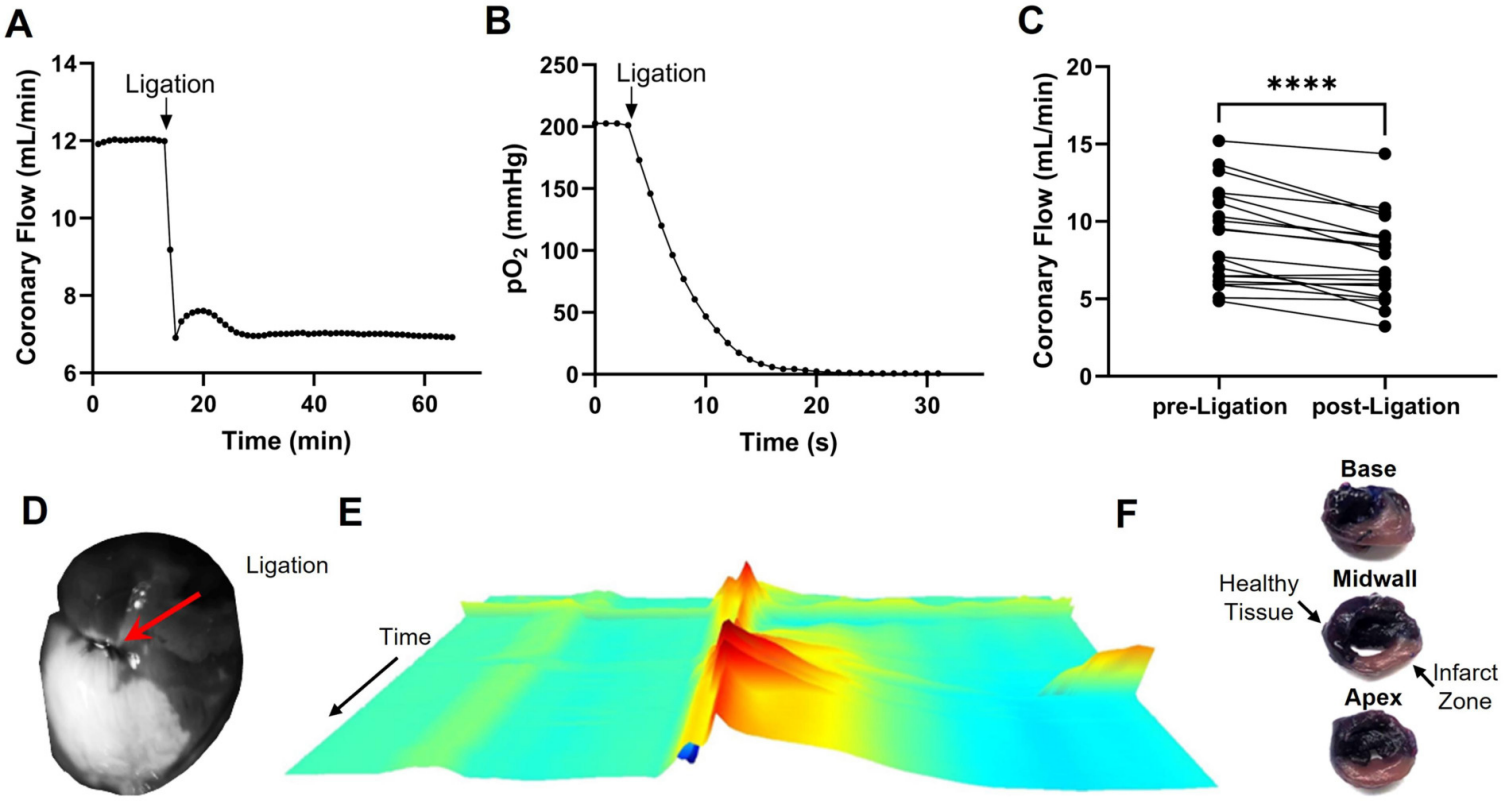
Outcomes of LAD ligation in perfused LE rat hearts. **(A)** Representative coronary flow measurement showing a reduction following LAD ligation. **(B)** Myocardial ${\mathrm{pO}}_{2}$ within the ischemic zone rapidly drops after LAD ligation. **(C)** Total coronary flow is significantly reduced after LAD ligation (two-tailed paired *t*-test, ****p<0.0001, *n* = 20). **(D)** NADH fluorescence image of the heart with red arrow showing the placement of the suture for LAD ligation. Bright white tissue is hypoxic, dark gray tissue is normoxic. **(E)** Waterfall ECG plot reveals ST-segment elevation after LAD ligation, consistent with myocardial ischemia. **(F)** TTC-stained slices of the base, midwall, and apex showing infarcted tissue (pink) and healthy myocardium (dark purple), highlighting transmural ischemia.

Ligation of the LAD resulted in a stable ischemic zone that encompassed a large portion of the LV with a well defined boundary, coinciding with a significant drop in total coronary flow. ${\mathrm{pO}}_{2}$ in the center of the ischemic zone also dropped to 0 mmHg within 10 s (Figure 3B). Instantaneous drops in total coronary flow were observed after LAD ligation (Figure 3A). On average, coronary flow decreased from 8.970 ± 3.099 mL/min at baseline to 7.578 ± 2.719 mL/min post-ligation (Figure 3C). Within 30 s after LAD ligation, NADH fluorescence revealed high contrast between ischemic (bright white) and normoxic (dark gray) tissue delineating a distinct boundary (Figure 3D). ST-segment elevation, an indicator of myocardial ischemia, was also observed (Figure 3E). The ischemic zone was transmural in all hearts (Figure 3F). The size of the myocardial area-at-risk did not change with DREADDs activation (No DREADDs: 40.46 ± 7.64, *n* = 6; DREADDs: 36.44 ± 8.09, *n* = 7, *p* = 0.3768). Although the area-at-risk encompassed approximately half of the LV, there was high variability in the area-at-risk size between all hearts, confounding the detection of differences in area-at-risk size between the hearts of rats that expressed DREADDS and those that did not.

### 3.3 PR interval and arrhythmias

**Figure 4:**
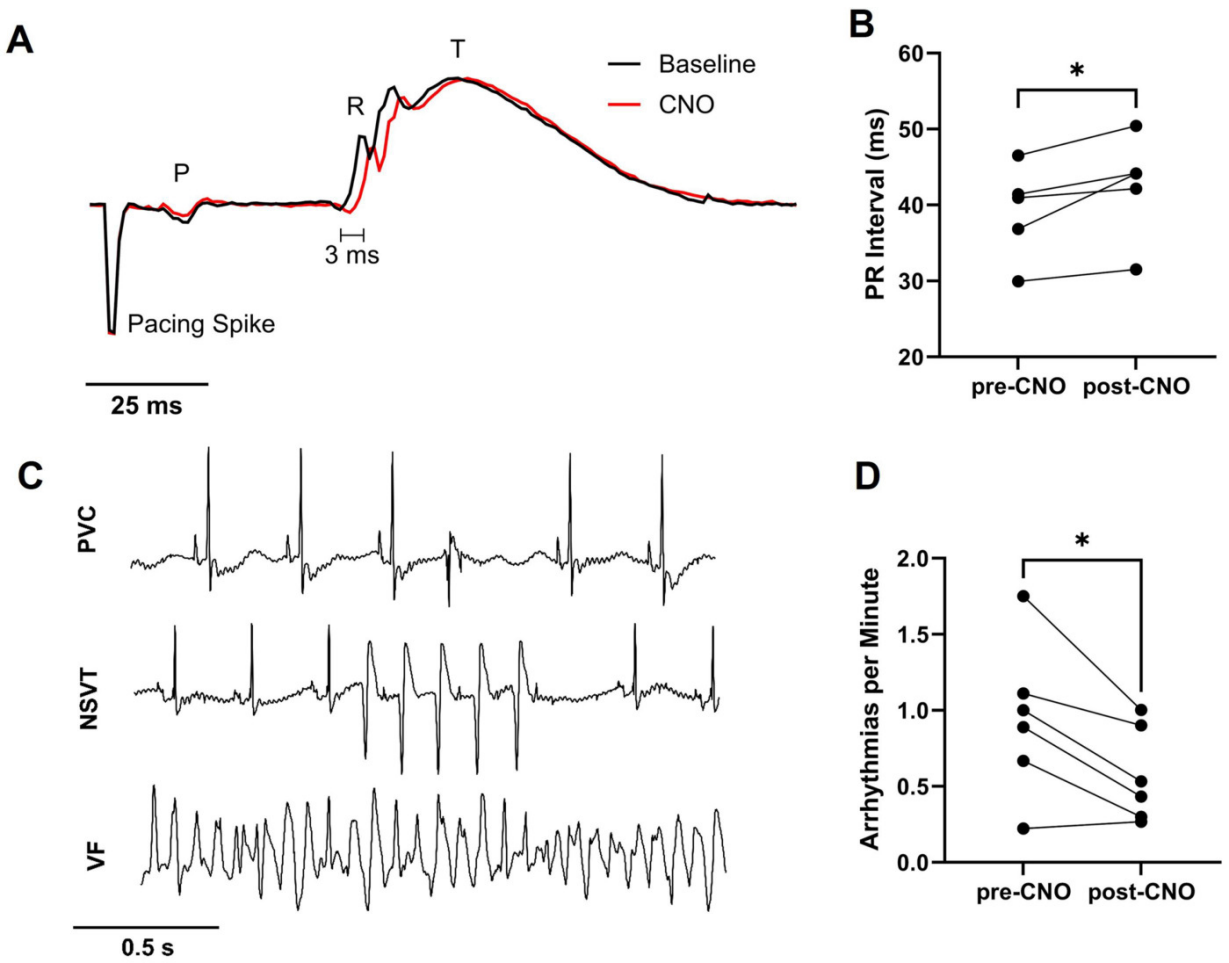
DREADDs activation of cholinergic ICG neurons *ex vivo* prolongs PR interval and reduces arrhythmias after LAD ligation. **(A)** Example ECG signal overlaying baseline (black) and a recording 10 min after CNO administration (red) highlighting delayed atrioventricular conduction. **(B)** Quantification of the PR interval duration before CNO treatment (pre-CNO) and 10 min after CNO treatment (post-CNO) demonstrates a significant lengthening following CNO administration in DREADDs-expressing rats (two-tailed paired *t*-test, *p* = 0.0378, *n* = 5). (C) Example arrhythmias observed after LAD ligation including PVCs, NSVT, and VF. **(D)** Arrhythmia incidence after LAD ligation but before treatment (pre-CNO) and after CNO administration (post-CNO). (Two-tailed paired *t*-test, *p* = 0.0199, *n* = 6).

After administering CNO, PR interval was prolonged from 39.13 ± 6.17 ms before CNO to 42.46 ± 6.87 ms (*p* = 0.0378, *n* = 5), indicating slowed AV conduction after DREADDs activation of cholinergic ganglia during ischemia (Figures 4A,B). Arrhythmias were commonly observed post-LAD ligation and included PVCs, NSVT, and VF (Figure 4C). Arrhythmia incidence significantly decreased after administering CNO (0.9398 ± 0.5063 $\min^{-1}$ to .5727 ± 0.3103 $\min^{-1}$; *p* = 0.0199, *n* = 6) (Figure 4D).

### 3.4 Border zone oxygenation

**Figure 5:**
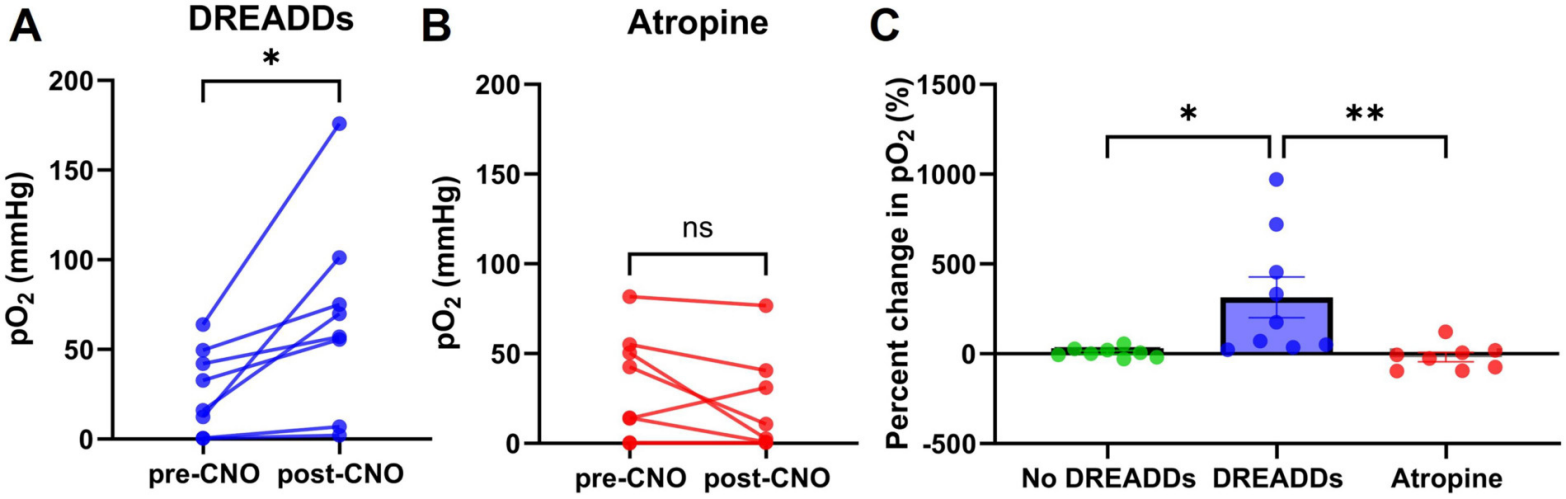
DREADDs activation of cholinergic ICG neurons increases ischemic border zone oxygenation. **(A)** ${\mathrm{pO}}_{2}$ in the infarct border zone of DREADDs-expressing rats was significantly higher after CNO administration (two-tailed paired *t*-test, *p* = 0.0131, *n* = 9). **(B)** Atropine prevented infarct border zone ${\mathrm{pO}}_{2}$ elevations after CNO administration (two-tailed *t*-test, *p* = 0.1391, *n* = 8). **(C)** Infarct border zone ${\mathrm{pO}}_{2}$ percent change was low for control (No DREADDs) hearts, highest for DREADDs-expressing (DREADDs) hearts, and low for DREADDs-expressing hearts that received atropine before CNO (Atropine). A one-way ANOVA with Tukey’s post hoc test determined statistical significance (No DREADDs vs. DREADDs, *p* = 0.0168; DREADDs vs. Atropine, *p* = 0.0095). Data are presented as mean ± SEM.

Cholinergic ICG neuron activation after LAD ligation increased border zone ${\mathrm{pO}}_{2}$ (42.13 ± 49.82 mmHg to 82.25 ± 66.87 mmHg), *p* = 0.0131, *n* = 9). Administering atropine before CNO prevented increases in border zone oxygenation (32.32 ± 29.59 mmHg to 20.35 ± 27.52 mmHg, *p* = 0.1391, *n* = 8) (Figures 5A,B). Percent change in ${\mathrm{pO}}_{2}$ was significantly higher in hearts from DREADDs-expressing rats compared to hearts without DREADDs (*p* = 0.0168, *n* = 8). Percent change in ${\mathrm{pO}}_{2}$ was significantly lower in hearts from DREADDs-expressing rats that received atropine (*p* = 0.0095, *n* = 8) and was no different than that of hearts from rats without DREADDs (Figure 5C).

**Figure 6:**
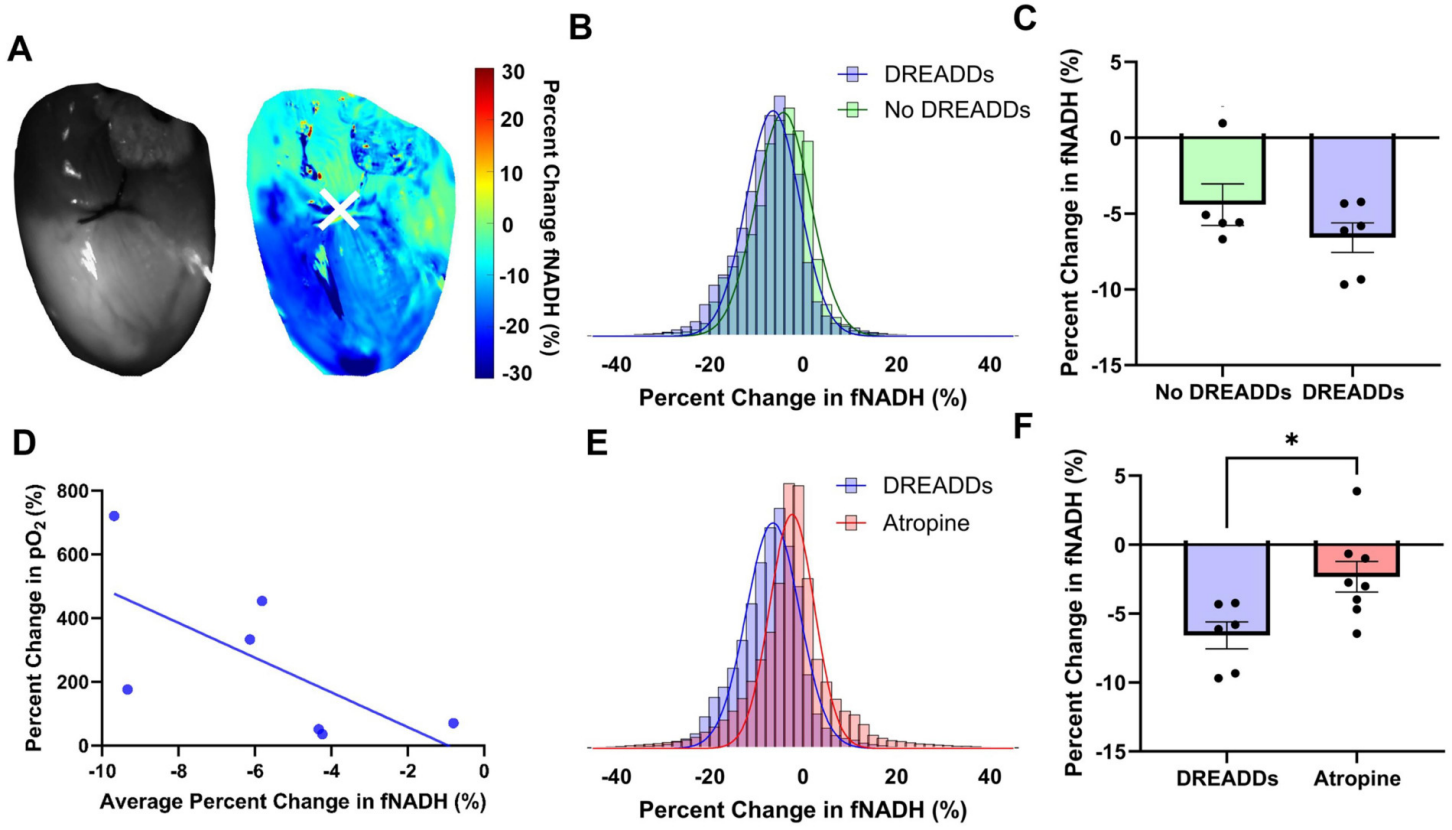
Changes in epicardial NADH fluorescence following DREADDs activation of cholinergic ICG neurons. **(A)** Raw image (left) and colormap (right) showing the spatial distribution of changes in fNADH 10 min after CNO administration for a DREADDs-expressing heart. The white “X” indicates the LAD suture. **(B)** Histograms comparing the percent change in fNADH after CNO administration to the hearts of DREADDs rats (*n* = 6) and no-DREADDs rats (*n* = 5). **(C)** Bar graph showing a trending decrease in fNADH after CNO administration to DREADDs hearts (*n* = 6) compared to no-DREADDs hearts (*n* = 5, Kolmogorov-Smirnov test). **(D)** Scatterplot illustrating that border zone ${\mathrm{pO}}_{2}$ decreases with increasing epicardial fNADH after CNO administration to DREADDs hearts (*n* = 7). **(E)** Histogram comparing the percent change in fNADH between DREADDs hearts (*n* = 6) and atropine-treated DREADDs hearts (*n* = 8). **(F)** Bar graph showing a significant decrease in fNADH after CNO administration to DREADDs hearts (*n* = 6) compared to atropine-treated DREADDs-expressing hearts (*n* = 8) (Two-tailed unpaired *t*-test, *p* = 0.0172, DREADDs *n* = 6, atropine *n* = 8). Bar graphs are presented as mean ± SEM.

After administering CNO, NADH oxidation had an increasing trend in hearts from DREADDs-expressing rats compared to hearts without DREADDs, measured as a percent decrease in fNADH (Figures 6B,C). As the percent change in ${\mathrm{pO}}_{2}$ increased, fNADH decreased (Figure 6D), confirming that tissue oxygenation was correlated with changes in epicardial fNADH. Administering atropine before CNO prevented increases in NADH oxidation, indicated by a lower percent decrease in fNADH compared to DREADDs-expressing hearts that did not receive atropine (*p* = 0.0172) (Figures 6E,F).

## 4 Discussion

Acute MI remains a leading cause of morbidity and mortality worldwide. Current therapeutic strategies rely on reperfusion and pharmacological management, which are typically administered hours after a cardiac event. Our previous work demonstrated that central activation of the cardiac cholinergic network at the level of the PVN soon after an MI decreased arrhythmia burden, attenuated inflammation, and decreased infarct size [19]. Building on these findings, this work demonstrates that direct activation of cholinergic ICG neurons 20 min after a coronary occlusion may also be cardioprotective. This identifies the cardiac autonomic ganglia as a therapeutic target for intervention before reperfusion therapy for early activation of cardioprotective pathways. Overall, our results support the development of neuromodulation-based therapies that can be initiated at the level of the heart during the early stages of an MI using device-based or pharmacological approaches that target the cholinergic neurons.

### 4.1 Cholinergic activation

Cholinergic neurons release acetylcholine (ACh) to decrease HR and increase atrioventricular node conduction time [23]. Reductions in HR after administering CNO to conscious unrestrained rats confirmed DREADDs activation of cholinergic ICG neurons *in vivo* (Figure 2). Prolonged PR interval after administering CNO to the perfused hearts of DREADDs-expressing rats further confirmed DREADDs activation of cholinergic neurons *ex vivo* (Figures 4A,B). The moderate degree of PR prolongation we observed is consistent with physiological vagal activation and falls below the thresholds associated with adverse clinical outcomes, which are primarily linked to marked or persistent PR prolongation [24, 25]. Atropine administration before CNO administration prevented increases in border zone oxygenation (Figure 5) and reversed the trend of increased NADH oxidation (Figure 6), confirming the involvement of muscarinic receptor activation downstream of cholinergic ICG activation and ACh release.

### 4.2 Vasodilation

The parasympathetic nervous system promotes arteriole vasodilation, including within the salivary glands [26] and the GI tract [27]. Cholinergic neuron release of ACh activates endothelium dependent vasodilation through muscarinic M3 receptor-mediated NO release [28, 29], which could be a mechanism of our observed increase in ischemic border zone oxygenation after DREADDs activation of cholinergic ICG neurons (Figure 5). This is supported by our observation that atropine prevented increased ischemic border zone oxygenation (Figure 5C) and prevented reductions of epicardial NADH fluorescence (Figure 6F) after cholinergic ICG activation. Another potential mechanism is the release of vasoactive intestinal peptide (VIP), a potent vasodilator, from VIP-containing nerve fibers located within the walls of the coronary arteries. Vagal stimulation, which activates cardiac preganglionic efferents, has been shown to release VIP to increase coronary flow [30] and promote local blood flow during acute myocardial ischemia [31]. Although ventricular cholinergic neuron innervation is considered to be sparse compared to that of atrial tissue [32], studies have shown that these neurons may have significant effects on ventricular contractility [33]. Additionally, dense innervation of the coronary vasculature by cholinergic neurons could allow the ICG network to directly regulate regional coronary flow.

### 4.3 Oxidation of ischemic border zone tissue

Although total coronary flow did not change following cholinergic ICG activation, we observed regional improvements in tissue oxygenation (${\mathrm{pO}}_{2}$) within border zone tissue (Figure 5). This suggests that cholinergic ICG activation may have induced localized redistribution of flow that did not alter total flow. The border zone lies between healthy and ischemic myocardium and is responsive to increased collateral flow through anastomotic connections [34, 35]. Enhanced recruitment of pre-existing coronary anastomoses has been shown to facilitate collateral circulation to the border zone, contributing to the improvements in local tissue oxygenation [36]. Coronary collateral circulation occurs through a network of pre-existing arteriolar anastomoses connecting adjacent vascular territories, which can be recruited and dilated in response to ischemia to redistribute blood flow [37, 38]. Although we did not measure local coronary flow, our observations are aligned with these mechanistic underpinnings of flow redistribution during local ischemia, which could be crucial for maintaining tissue viability in the border zone.

### 4.4 Collateral flow

Rats have limited pre-existing coronary anastomoses and reduced native collateral microcirculation reserve compared to species such as guinea pigs and dogs, which have extensive coronary anastomotic networks [39]. While this species difference limits the magnitude of collateral flow recruitment that is possible in perfused rat hearts, the presence of any anastomotic connections allows for regional flow redistribution following parasympathetic stimulation. Additionally, crystalloid-perfused hearts exhibit baseline coronary vasodilation to compensate for the limited oxygen-carrying capacity of perfusate solutions [40, 41]. As a result, the baseline coronary flow of perfused rodent hearts are hyperphysiologic, having approximately 50%–60% of maximally dilated flow [42, 43]. This baseline vasodilation lowers coronary flow reserve, potentially masking vasodilatory responses to cholinergic ICG activation, such that the effect of cholinergic ICG activation could be greater *in vivo* than what we observed in our perfused heart studies.

### 4.5 Arrhythmias

Elevation of parasympathetic tone is therapeutic for a wide range of cardiac disease states [44], including reducing arrhythmia vulnerability during acute coronary occlusions [19, 45] and healed myocardial infarctions [12, 46, 47]. Our results further demonstrate that selective activation of cholinergic ICG neurons significantly reduces arrhythmias after a coronary occlusion, with reduced incidence of PVCs, NSVT, and VF (Figure 4). In addition to the beneficial effects of vasodilation, negative chronotropy, and negative inotropy on the myocardium during ischemia, cholinergic activation of muscarinic pathways suppresses beta-adrenergic signaling [48] to reduce sympathetic-mediated elevation of ventricular excitability and arrhythmia burden [49]. These beneficial effects of ACh during acute ischemic injury are also provided by elevated vagal activity and vagal stimulation [47, 50]. ACh also reduces the automaticity of Purkinje fibers [51], which have been implicated as a source of ventricular arrhythmias during ischemia [52, 53]. Suppression of beta-adrenergic signaling and reduced Purkinje automaticity are two potential mechanisms of reduced arrhythmia burden that we observed when activating cholinergic ICG neurons during a coronary occlusion.

### 4.6 Limitations

Limitations include those of typical perfused heart experiments, including the lack of erythrocytes and circulating hormones [41, 54]. Acute LAD ligation in the rat also does not fully recapitulate the complexity of a human MI due to differences in vasculature [55], collateral circulation [39], and autonomic control [11]. Our short-term heart perfusion studies focused on the effect of acute activation of the cholinergic ICG neurons after an acute ischemic event. Long-term effects of cholinergic ICG neuron activation on infarct size and cardiac remodeling were not studied. Measurements of changes in epicardial fNADH and local tissue ${\mathrm{pO}}_{2}$ support the premise that perfusion of ischemic border zone tissue improved after cholinergic ICG neuron activation. However, changes in local tissue perfusion were not measured and will be pursued in future studies. Elevation of parasympathetic tone could also slow HR, cause AV block, and shorten action potential duration in the atria to promote atrial arrhythmias [56].

### 4.7 Conclusions

Chemogenetic activation of cholinergic ICG neurons improved border zone oxygenation and reduced arrhythmia incidence shortly after LAD ligation in perfused rat hearts, demonstrating that selective stimulation of cardiac cholinergic neurons could activate cardioprotective pathways soon after an ischemic event. Our results support the premise that cell-specific stimulation of intrinsic cardiac cholinergic neurons shortly after a coronary occlusion is a promising therapeutic approach that addresses the limitations of conventional vagal stimulation.

## Data Availability

The raw data supporting the conclusions of this article will be made available by the authors, without undue reservation.
